# Association Between Initial Symptoms and Clinical Outcomes in COVID-19

**DOI:** 10.7759/cureus.84919

**Published:** 2025-05-27

**Authors:** Eiki Ichihara, Toshiharu Mitsuhashi, Mitsuru Tsuge, Kou Hasegawa, Kenichiro Kudo, Yasushi Tanimoto, Kazuhiro Nouso, Naohiro Oda, Sho Mitsumune, Goro Kimura, Haruto Yamada, Ichiro Takata, Hideharu Hagiya, Akihiko Taniguchi, Kohei Tsukahara, Toshiyuki Aokage, Shinichi Toyooka, Hirokazu Tsukahara, Yoshinobu Maeda

**Affiliations:** 1 Department of Allergy and Respiratory Medicine, Okayama University Hospital, Okayama, JPN; 2 Center for Innovative Clinical Medicine, Okayama University Hospital, Okayama, JPN; 3 Department of Pediatrics, Okayama University Graduate School of Medicine, Dentistry, and Pharmaceutical Sciences, Okayama, JPN; 4 Department of General Medicine, Okayama University Graduate School of Medicine, Dentistry, and Pharmaceutical Sciences, Okayama, JPN; 5 Department of Respiratory Medicine, National Hospital Organization (NHO) Okayama Medical Center, Okayama, JPN; 6 Department of Gastroenterology, Okayama City Hospital, Okayama, JPN; 7 Department of Internal Medicine, Fukuyama City Hospital, Fukuyama, JPN; 8 Department of Infectious Diseases, Okayama City Hospital, Okayama, JPN; 9 Department of Emergency, Critical Care, and Disaster Medicine, Okayama University Graduate School of Medicine, Dentistry, and Pharmaceutical Sciences, Okayama, JPN; 10 Department of Emergency Medicine, Tokyo Metropolitan Institute for Geriatrics and Gerontology, Tokyo, JPN; 11 Department of General Thoracic Surgery and Breast and Endocrinological Surgery, Okayama University Graduate School of Medicine, Dentistry, and Pharmaceutical Sciences, Okayama, JPN; 12 Department of Hematology, Oncology, and Respiratory Medicine, Okayama University Graduate School of Medicine, Dentistry, and Pharmaceutical Sciences, Okayama, JPN

**Keywords:** clinical outcome, covid-19, localized respiratory symptom, severe disease risk, systemic symptom

## Abstract

Background: The clinical presentation of coronavirus disease 2019 (COVID-19) ranges from localized respiratory symptoms such as cough and sore throat to systemic symptoms such as fever and fatigue. To our knowledge, no study has assessed severe disease risk by dividing onset symptoms into localized respiratory and other symptoms. We aimed to determine whether the risk of severe COVID-19 differs depending on whether the symptoms at onset are limited to local respiratory symptoms.

Method: This was a multicenter prospective cohort study. The patients were classified into localized respiratory or systemic symptom groups based on the symptoms at onset. Demographic data, blood biomarkers, and clinical outcomes, including mortality, intubation, admission to the intensive care unit, and time to discharge, were compared. This study included 100 adult patients diagnosed with COVID-19 between July 2020 and August 2021.

Result: Twelve patients were classified into the localized respiratory symptom group and the remaining 88 into the systemic symptom group. No significant differences between the groups were observed in the baseline characteristics, blood biomarkers, or clinical outcomes. The mortality rates were 0.0% and 4.6%, respectively. The median durations to discharge were 11 and 10 days, respectively (p=0.512). The levels of inflammatory and oxidative stress biomarkers, including interleukin-6 and hydroperoxides, were similar between the groups.

Conclusion: The symptom type at disease onset was not significantly associated with differences in clinical outcomes. Comprehensive assessments beyond initial symptoms are crucial for predicting disease progression and optimizing management strategies.

## Introduction

Coronavirus disease 2019 (COVID-19), caused by the novel severe acute respiratory syndrome coronavirus 2 (SARS-CoV-2), emerged in late 2019 and quickly spread globally. The clinical presentation of COVID-19 is highly variable, ranging from asymptomatic infection to severe respiratory failure and multiorgan dysfunction [1]. At disease onset, some patients present with localized respiratory symptoms (LRS) such as sore throat and cough, whereas others show systemic symptoms (SS) including fever, fatigue, and myalgia [2]. This wide variation in initial symptoms suggests that the pathophysiology and immune responses to SARS-CoV-2 infection differ across individuals. Understanding how the initial symptoms relate to patient outcomes is essential for effective clinical management and resource allocation. The nature of the early symptoms may offer crucial insights into the course of the disease and its prognosis.

Several studies have demonstrated that factors such as age and preexisting conditions significantly influence the severity and prognosis of COVID-19 [3]. However, limited research has specifically addressed how the nature of the initial symptoms, localized versus systemic, affects disease progression and clinical outcomes. LRS, such as sore throat and cough, may indicate a milder form of the disease, whereas SS, such as fever, fatigue, and myalgia, could reflect a more widespread immune response and potentially lead to more severe disease progression [4]. Moreover, early recognition of these distinctions could help identify high-risk patients and improve resource distribution.

Understanding these differences in clinical presentation can guide public health strategies and inform therapeutic decisions. For example, patients with SS may benefit from proactive monitoring and earlier interventions. By differentiating between LRS and SS, this study aimed to provide valuable insights that could aid in the early stratification of patients and help tailor treatment strategies. This study addresses a critical gap in the literature and provides important information for managing the diverse clinical manifestations of COVID-19.

The primary objective of this study was to explore whether the demographic, clinical, and prognostic characteristics of patients with LRS at the onset of COVID-19 differ from those presenting with SS. By examining these differences, we aimed to gain a better understanding of how the initial symptomatology, specifically the distinction between LRS and SS, affects the subsequent clinical course, treatment needs, and outcomes of COVID-19, thereby offering novel insights into patient stratification and early risk assessment. This knowledge is essential for the development of personalized clinical management strategies and for improving patient care in future respiratory disease outbreaks.

## Materials and methods

Trial design

This multicenter, observational, prospective cohort study was performed as part of a phase II randomized controlled clinical trial investigating the efficacy of teprenone in patients with COVID-19 [5]. A total of 100 adult Japanese patients diagnosed with COVID-19 between July 2020 and August 2021 were prospectively included in this study. The design of this study has been described previously [5]. This study was approved by the Certified Review Board of Okayama University (CRB20-001) on April 28, 2020, and registered in the Japan Registry of Clinical Trials (jRCTs061200002) on May 20, 2020. The study was conducted in compliance with the principles of the Declaration of Helsinki, and informed consent was obtained from all patients before any screening procedure or inclusion. The participants were recruited from July 15, 2020, to August 18, 2021. Each patient was followed up for 10 days or until discharge. The rationale for establishing a 10-day follow-up period is that the study was conducted during the early phase of the COVID-19 pandemic in 2020. During that period, the isolation period in Japan was, in principle, at least 10 days from the onset of symptoms.

Patients

The inclusion criteria were age ≥20 years at the time of enrollment, diagnosis of COVID-19 confirmed by polymerase chain reaction or an antigen test with fever of ≥37.5°C, and provision of written informed consent. All patients were hospitalized in either a hospital or a comparable facility, as it was obligatory in Japan to hospitalize all individuals diagnosed with COVID-19 during the study period. The all-age COVID-19 vaccination program was launched in Japan in June 2021 during the study period, and the last four patients were enrolled thereafter.

Statistical analyses

Descriptive statistics included medians and ranges for continuous variables and frequencies and percentages for categorical variables. This analysis also evaluated the rates of mortality, intubation, and intensive care unit (ICU) admission, which are the primary or secondary endpoints of the main analyses in this clinical trial [5]. Mortality outcomes between groups were assessed using the Cox proportional hazards model. For events such as intubation and admission to the ICU, where death represented a competing event, subhazard ratios (SHRs) were determined using the Fine and Gray method [6]. In the analyses involving time to oxygen discontinuation and time to hospital discharge, death was treated as a competing risk. Cumulative incidence curves accounting for competing risks were generated, and group comparisons were performed using the Pepe and Mori test [6,7]. To adjust for confounding factors, a stabilized weight was calculated based on the propensity score and adjusted SHR, and odds ratios (OR) were determined for each outcome. Propensity scores were calculated using a logistic regression model in which the dependent variable was the onset of symptoms and the independent variables included potential confounding factors. Stabilized weights were computed by dividing the marginal probability of symptoms at onset by the conditional probability (i.e., propensity score). The balance of confounders was assessed using standardized mean differences (SMDs), considering values below 0.1 as acceptable, and variance ratios (VRs) between 0.8 and 1.25 were used to confirm the balance [8,9]. The confounding factors included age, sex, body mass index (BMI), smoking history, hypertension, diabetes, cardiovascular disease, and chronic pulmonary disease (COPD). The propensity score method was applied after confirming the presence of common support between groups. All statistical analyses were conducted using Stata 18/MP8 software (StataCorp LLC, College Station, TX, USA). Statistical significance was set at p<0.05. Because this was an exploratory study, no adjustments were made for multiple comparisons [10].

Measurement of blood samples

Blood samples were obtained at the time of hospitalization. Lymphocyte counts and serum levels of lactate dehydrogenase (LDH), Krebs von den Lungen-6 (KL-6), ferritin, D-dimer, and C-reactive protein (CRP) were analyzed in clinical practice settings at each participating hospital. Measurements of interleukin-6 (IL-6), asymmetric dimethylarginine, and arginase-1 were performed using enzyme-linked immunosorbent assay kits following the protocols specified by the manufacturers, as previously described [11,12]. The following kits were used: IL-6 (R&D Systems, Inc., Minneapolis, MN, USA), asymmetric dimethylarginine (Immundiagnostik AG, Manchester, NH, USA), and arginase-1 (RayBiotech, Inc., Peachtree Corners, GA, USA). The hydroperoxide concentrations in the serum were assessed using a diacron-reactive oxygen metabolite (d-ROM) kit on a Free Radical Analytical System (FREE Carrio Duo, Wismerll Co., Ltd., Tokyo, Japan) [13]. This method is based on the Fenton and Haber-Weiss reactions, with one unit of hydroperoxide (Carratelli unit (U. CARR)) corresponding to 0.08 mg/dL of H₂O₂. Nitrite/nitrate concentrations were determined using the Griess reaction, following the manufacturer's instructions (R&D Systems, Inc., Minneapolis, MN, USA). Serum levels of arginine, citrulline, ornithine, and ammonia were quantified using high-performance liquid chromatography performed in a commercial laboratory (Biomedical Laboratories, Tokyo, Japan). Due to sample volume limitations in certain cases, amino acid measurements were obtained from 79 patients.

## Results

Patient characteristics

A total of 100 patients from five institutions were included in this study. All patients were classified into either the LRS group, defined as those who presented only with LRS, such as sore throat and cough, or the SS group, defined as those who presented with any SS, such as fever and fatigue. Among the patients included in the study, 12 were classified into the LRS group, whereas 88 were classified into the SS group. The patient characteristics are listed in Table 1. The characteristics of the two groups were similar. The median ages of the LRS and SS groups were 71 and 63 years, respectively, and the BMIs were 22.7 and 23.8 kg/m², respectively. The male-to-female ratio was almost equal in the LRS group, whereas the SS group consisted of more males. Half of the patients in both groups had a history of smoking. Similar frequencies of coexisting conditions, including hypertension, diabetes, cardiovascular disease, COPD, and active malignant neoplasms, were observed in both groups.

**Table 1:** Patient characteristics

Variables	Systemic (n=88)	Localized (n=12)
Age (years) median (range)	63 (21–88)	71 (31–81)
Body mass index (kg/m²) median (range)	23.8 (15.2–42.0)	22.7 (18.9–37.2)
Gender		
Female	33 (37%)	7 (58%)
Male	55 (63%)	5 (42%)
Smoking history		
Yes	43 (49%)	6 (50%)
No	38 (43%)	5 (42%)
Unknown	7 (8%)	1 (8%)
Coexisting conditions		
Hypertension	29 (33%)	6 (50%)
Diabetes	17 (19%)	3 (25%)
Cardiovascular disease	4 (8%)	1 (8%)
Chronic pulmonary disease	6 (7%)	1 (8%)
Active malignant neoplasm	2 (2%)	0 (0%)

Blood test findings between the groups

The blood test results were compared between the groups, as shown in Table 2. Laboratory parameters such as lymphocyte count, LDH, KL-6, ferritin, D-dimer, and CRP were similar between the LRS and SS groups: lymphocyte count 1,050 vs. 970/μL; LDH 247 vs. 272 U/L; KL-6 229 vs. 206 U/mL; ferritin 681 vs. 696 ng/mL; D-dimer 0.7 vs. 0.3 μg/mL; and CRP 2.2 vs. 1.5 mg/dL. Additionally, oxidative stress-related biomarkers and serum amino acid levels were analyzed (Table 2). Our previous research indicated that elevated oxidative stress markers and reduced citrulline metabolism are linked to severe COVID-19 [14]. The levels of the following markers were also similar between the groups: interleukin-6 30 vs. 28 pg/mL, hydroperoxides 30 vs. 32 mg/dL, nitrite/nitrate 26 vs. 31 µmol/L, asymmetric dimethylarginine 0.27 vs. 0.10 µmol/L, arginine 134 vs. 136 nmol/mL, ornithine 81 vs. 99 nmol/mL, citrulline 22 vs. 23 nmol/mL, ammonia 115 vs. 142 nmol/mL, and arginase-1 155 vs. 203 ng/mL.

**Table 2:** Blood test findings of the groups * Due to limitations in sample volume, IL-6, hydroperoxides, nitrite/nitrate, asymmetric dimethylarginine, arginine, ornithine, citrulline, ammonia, and arginase-1 were measured in 79 out of 100 patients (68 from the systemic group and 10 from the localized group). LDH: lactate dehydrogenase, KL-6: Krebs von den Lungen-6, CRP: C-reactive protein, IL-6: Interleukin-6

Parameters	Normal Range	Systemic (n=88*)	Localized (n=12*)
Lymphocyte, /μL	Not determined	1,050 (778–1,412)	970 (715–1,165)
LDH, U/L	124-222	247 (204–330)	272 (194–348)
KL-6, U/mL	< 500	229 (178–295)	206 (178–428)
Ferritin, ng/mL	39.9-465	681 (291–1,978)	696 (234–760)
D-dimer, μg/mL	0.0-0.9	0.7 (0.0–1.2)	0.3 (0.0–1.9)
CRP, mg/dL	< 0.15	2.2 (0.4–6.6)	1.5 (0.3–4.1)
IL-6, pg/mL	< 7.0	30 (16–62)	28 (17–88)
Hydroperoxides, mg/dL	Not determined	30 (26–33)	32 (28–34)
Nitrite/nitrate, µmol/L	Not determined	26 (18–36)	31 (30–33)
Asymmetric dimethylarginine, µmol/L	Not determined	0.27 (0.13–0.42)	0.10 (0.05–0.23)
Arginine, nmol/mL	31.8-149.5	134 (111–150)	136 (109–143)
Ornithine, nmol/mL	42.6-141.2	81 (66–99)	99 (60–133)
Citrulline, nmol/mL	17.9-48.0	22 (17–29)	23 (19–30)
Ammonia, nmol/mL	71.8-230.4	115 (100–138)	142 (129–180)
Arginase-1, ng/mL	Not determined	155 (110–233)	203 (137–276)

Association between clinical outcomes and symptoms at onset

Several variables showed an imbalance before weighting with stabilized weights (Supplementary Material). After weighting, variables including smoking history, COPD, and active cancer exhibited SMD and VR values outside the acceptable range. However, the overall balance of confounding factors improved with SMD and VR.

**Figure 1:**
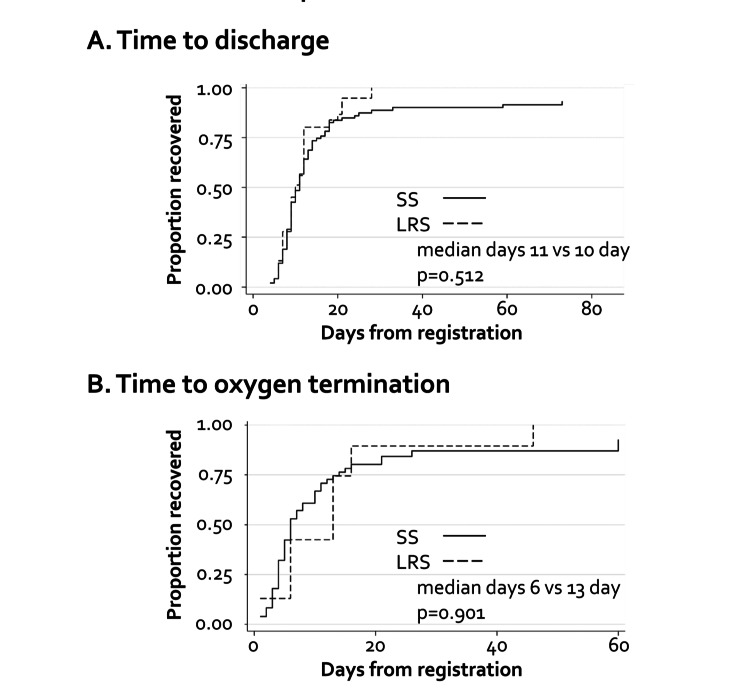
Times to clinical improvement (A) Time to discharge; (B) time to supplementary oxygen termination. SS: systemic symptoms, LRS: localized respiratory symptoms

Subsequently, we compared the clinical outcomes between the groups. The rates of mortality, intubation, and ICU admission were determined for each group (Table 3). The hazard ratio (HR) was not determined for mortality because no deaths had occurred in the LRS group; the mortality rates were 4.6% in the SS group and 0.0% in the LRS group. For intubation and ICU admission, we calculated the SHR instead of conventional HR because death represented a competing event. No significant differences were observed in the intubation or ICU admission rates. For intubation, the SHR was 0.72 (95% CI: 0.08-5.56; p = 0.68), and for ICU admission, the SHR was 0.44 (95% CI: 0.08-2.23; p = 0.33). The rate of need for oxygen inhalation of more than 5 L/minute also did not differ between the groups (17.1 vs. 16.7%, p=0.91) (Table 4). The time to clinical improvement showed no significant between-group differences, with a median time to discharge of 11 vs. 10 days (SHR: 0.80, 95% CI: 0.50-1.28, Pepe and Mori test p = 0.512) (Figure 1A) and a median time to oxygen termination of six vs. 13 days (SHR: 1.07, 95% CI: 0.58-1.99, Pepe and Mori test p = 0.901) (Figure 1B).

**Table 3:** Severity rate * Fine and Gray method. HRs were not calculated for mortality because there were no deaths in localized respiratory symptom groups. For the other failure events, sub-hazard ratios were calculated with death as a competing event. CI: confidence interval, ICU: intensive care unit, HR: hazard ratio, SHR: sub-hazard ratio

Variables	Systemic (n=88)	Localized (n=12)	Adjusted SHR
Mortality			
% (n)	4.6% (4)	0.0% (0)	N/A
95% CI	1.2–11.2%	0.0–22.1%	N/A
Intubation			
% (n)	5.7% (5)	8.3% (1)	0.72
95% CI	0.1–10.9%	3.2–21.4%	0.08–5.56
p*			0.68
Admission to ICU			
% (n)	6.8% (6)	25.0% (3)	0.44
95% CI	2.5–14.3%	5.5–57.2%	0.08–2.23
p*			0.334

**Table 4:** Maximum oxygen demand * Logistic regression analysis. OR: odds ratio, CI: confidence interval

Variables	Systemic (n=88)	Localized (n=12)	Adjusted OR
Maximum oxygen demand >5 L/min			
% (n)	17.1% (15)	16.7% (2)	0.91
95% CI	9.9–26.6%	2.1–48.4%	0.17–4.95
p*			0.91

## Discussion

Our study revealed that LRS were not necessarily associated with favorable outcomes in COVID-19 patients. We found no statistically significant differences between the two groups in terms of clinical outcomes such as mortality, intubation, ICU admission rates, or time to discharge. These findings suggest that it is essential to remain vigilant and consider the possibility of severe disease progression, even when the initial symptoms are limited to LRS.

Some studies have examined the association between individual symptoms and the risk of severe disease [15]; however, to our knowledge, no study has evaluated the risk of severe disease by classifying the symptoms at onset into LRS and other symptoms in patients with COVID-19. A meta-analysis found that cough, an LRS, was a risk factor for severe disease and SS, such as fever and fatigue; however, sore throat, another LRS, was not [16]. Regarding the severity of COVID-19, the nature of individual symptoms may be more important than whether the symptoms remain localized to the respiratory tract.

Elevated levels of inflammatory markers, such as LDH, KL-6, ferritin, D-dimer, CRP, and IL-6, as well as decreased lymphocyte counts, have been reported to be associated with poor outcomes in patients with COVID-19 [17,18]. Furthermore, we previously investigated the correlation of oxidative stress and blood amino acids with nitric oxide metabolism [14], and patients who required oxygen therapy had higher serum levels of hydroperoxides and lower levels of citrulline. In the current study, we conducted a comprehensive analysis that included not only well-known biomarkers associated with the clinical course of COVID-19, such as lymphocyte count, LDH, KL-6, ferritin, D-dimer, CRP, and IL-6, but also inflammatory biomarkers whose significance has not been fully elucidated, including hydroperoxides, nitrite/nitrate, asymmetric dimethylarginine, arginine, ornithine, citrulline, ammonia, and arginase-1. The levels of inflammatory and oxidative stress markers, including IL-6, hydroperoxides, and nitrite/nitrate, were comparable between the two groups, indicating that the underlying inflammatory response might not differ substantially based on the initial symptomatology. Additionally, the absence of significant disparities in patient characteristics, including age, BMI, and comorbidities such as hypertension and diabetes, further supports the notion that these factors, rather than whether the initial symptoms are localized or not, might play a more critical role in determining disease severity. Several studies have emphasized the impact of advanced age and preexisting conditions on COVID-19 outcomes [19-21].

Similar to our findings, a meta-analysis of studies on the predictive value of early symptoms in COVID-19 reported no significant association between initial COVID-19 disease severity and common persistent symptoms, except for dyspnea and fatigue [15]. These data underscore the multifactorial nature of COVID-19 progression, where the initial symptomatology may only partially reflect the underlying disease mechanisms.

This study has important implications for clinical practice. Given the absence of significant differences in the outcomes, it may not be necessary to stratify hospitalized COVID-19 patients solely based on their initial symptoms. Instead, comprehensive risk assessment tools incorporating demographic, clinical, and biomarker data may allow a more accurate prediction of the disease trajectory. Additionally, our findings support the utility of universal clinical protocols rather than symptom-specific interventions, potentially simplifying resource allocation in high-burden settings.

This study had some limitations. First, the study was conducted during the early phases of the COVID-19 pandemic. During this period, the SARS-CoV-2 Alpha and Delta strains, in addition to the original Wuhan strain, were dominant, and the currently dominant strains, such as KP3.1.1 and XEC, did not appear. Moreover, most patients in this study were enrolled before the initiation of the all-age COVID-19 vaccination program in June 2021 and were presumed unvaccinated. Owing to differences in the prevalent strains of COVID-19 and the status of vaccine development, the mortality rate (4.0%) observed in this study was higher than the current COVID-19 mortality rate of <1%. Secondly, this study did not include asymptomatic patients because they may have remained undiagnosed. Third, the propensity score weights did not fully balance all confounders, leaving some residual biases. Smoking history, COPD, and active cancer may overestimate the measures. However, the SHR and OR remained below 1, indicating that the conclusion holds even after accounting for this potential bias. Fourth, blood sample testing for some inflammatory and oxidative stress biomarkers was not available for all patients (available in 79 out of 100 patients). This may have introduced some bias into the analysis. Finally, only 12 patients were included in the LRS group. Undeniably, the small number of patients resulted in a lack of significant differences between the two groups, and the absence of a statistically significant difference in clinical outcomes does not necessarily indicate that the two groups had equivalent outcomes. Considering these limitations, our results should be interpreted with caution. Further studies are required to address these issues.

## Conclusions

In our cohort study, the symptom type at disease onset was not significantly associated with differences in clinical outcomes. Therefore, the risk of severe COVID-19 cannot be excluded even if the initial symptoms are localized to the respiratory tract. Comprehensive assessment using clinical and laboratory data remains essential for accurate risk stratification and management.

## Appendices

Supplementary Material 

**Table 5:** Weighted and unweighted SMD/VR Variables were considered well-balanced if SMD < 0.1 and 0.8 < VR < 1.25. Weighted: standardized weighting, SMD: absolute value of standardized mean difference, VR: variance ratio

Variables	Unweighting SMD/VR	Weighting SMD/VR
Age	0.42/0.89	0.02/0.91
Body mass index	0.15/0.68	0.10/0.71
Gender	0.42/0.89	0.02/0.93
Smoking history		
Yes	0.02/0.93	0.10/0.93
No	0.03/0.94	0.21/1.03
Unknown	0.01/0.89	0.16/0.61
Coexisting conditions		
Hypertension	0.34/0.82	0.08/0.98
Diabetes	0.13/0.77	0.02/0.95
Cardiovascular disease	0.01/0.89	0.04/0.83
Chronic pulmonary disease	0.06/0.77	0.14/1.70
Active malignant neoplasm	0.21/N/A	0.21/N/A
